# Pr_5_Si_3_N_9_

**DOI:** 10.1107/S1600536809016638

**Published:** 2009-05-14

**Authors:** Saskia Lupart, Wolfgang Schnick

**Affiliations:** a Department Chemie und Biochemie, Ludwig-Maximilians-Universität München, Lehrstuhl für Anorganische Festkörperchemie, Butenandtstrasse 5–13, D-81377 München, Germany

## Abstract

Single crystals of Pr_5_Si_3_N_9_, penta­praseodymium trisilicon nona­nitride, were obtained by the reaction of elemental praseo­dymium with silicon diimide in a radio-frequency furnace at 1873 K. The crystal structure consists of a chain-like Si—N substructure of corner-sharing SiN_4_ tetra­hedra. An additional *Q* ^1^-type [SiN_4_] unit is attached to every second tetra­hedron directed alternately in opposite directions. The resulting branched chains inter­lock with each other, building up a three-dimensional structure. The central atoms of the *Q* ^1^-type [SiN_4_] unit and of its attached tetra­hedron are situated on a mirror plane, as are two of the four crystallographically unique Pr^3+^ ions. The latter are coordinated by six to ten N atoms, with Pr—N distances similar to those of other rare earth nitridosilicates.

## Related literature

For isotypic compounds *Ln*_5_Si_3_N_9_ (*Ln* = La, Ce), see: Schmolke *et al.* (2009 ▶). For experimental details, see: Schnick & Huppertz (1997 ▶); Schnick *et al.* (1999 ▶). Typical atomic distances for rare earth nitridosilicates have been reported by Schnick (2001 ▶) and Lissner & Schleid (2004 ▶).

## Experimental

### 

#### Crystal data

Pr_5_Si_3_N_9_
*M* *_r_* = 914.91
Orthorhombic, 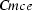

*a* = 10.512 (2) Å
*b* = 11.243 (2) Å
*c* = 15.773 (3) Å
*V* = 1864.2 (6) Å^3^
*Z* = 8
Mo *K*α radiation
μ = 26.01 mm^−1^
*T* = 293 K
0.17 × 0.10 × 0.08 mm

#### Data collection

Stoe IPDS diffractometer
Absorption correction: multi-scan (*XPREP*; Sheldrick, 2008 ▶) *T*_min_ = 0.040, *T*_max_ = 0.125
9626 measured reflections
1480 independent reflections
1133 reflections with *I* > 2σ(*I*)
*R*_int_ = 0.080

#### Refinement

*R*[*F* ^2^ > 2σ(*F* ^2^)] = 0.037
*wR*(*F* ^2^) = 0.097
*S* = 0.97
1480 reflections
90 parameters
Δρ_max_ = 2.10 e Å^−3^
Δρ_min_ = −2.31 e Å^−3^

### 

Data collection: *X-AREA* (Stoe & Cie, 2002 ▶); cell refinement: *X-AREA*; data reduction: *X-AREA*; program(s) used to solve structure: *SHELXS97* (Sheldrick, 2008 ▶); program(s) used to refine structure: *SHELXL97* (Sheldrick, 2008 ▶); molecular graphics: *DIAMOND* (Brandenburg, 1999 ▶); software used to prepare material for publication: *SHELXL97*.

## Supplementary Material

Crystal structure: contains datablocks global, I. DOI: 10.1107/S1600536809016638/wm2229sup1.cif

Structure factors: contains datablocks I. DOI: 10.1107/S1600536809016638/wm2229Isup2.hkl

Additional supplementary materials:  crystallographic information; 3D view; checkCIF report

## Figures and Tables

**Fig. 1.**
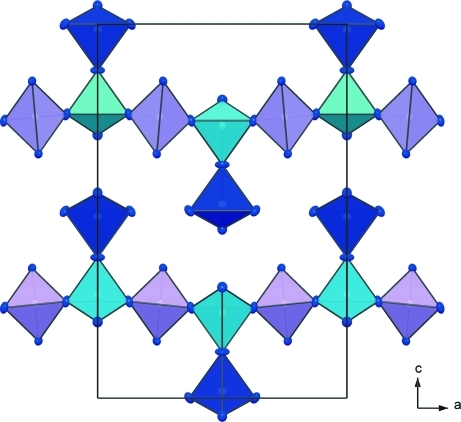
Presentation of the Si—N substructure, with anisotropic displacement parameters drawn at the 50% probability level. SiO4 tetrahedra are depicted purple for Si1, light blue for Si2 and dark blue for Si3.

**Fig. 2.**
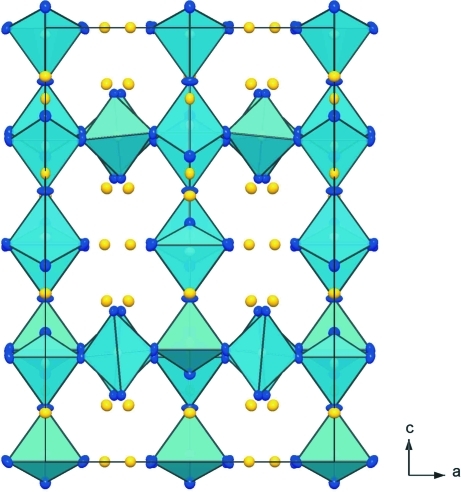
View along [010] illustrating the resulting three-dimensional structure. Pr3+ ions are depicted yellow, with anisotropic displacement parameters drawn at the 50% probability level.

## supplementary crystallographic information

### Comment

The title compound is a branched chain-like nitridosilicate isotypic to Ln_5_Si_3_N_9_ (Ln = La, Ce) described by Schmolke *et al.* (2009). Except for Ln_5_Si_3_N_9_ (Ln = La, Ce, Pr), no other chain-like nitridosilicates have been observed so far. The single chains in Pr_5_Si_3_N_9_ run along [100] and are built up of corner sharing [SiN_4_] tetrahedra, whereas every second tetrahedron is additionally connected to a Q^1^-type tetrahedron. These direct alternately in opposing directions (Fig. 1). Thereby the Si2 and Si3 atoms are located on a mirrow plane which is co-planar to [100]. Due to the constitution of the terminal tetrahedra the chains interlock zipper-like with each other (Fig. 2). The Pr^3+^ ions (yellow) are located between the chains. The coordination numbers of the Pr^3+^ ions range between six (for Pr4) and ten (for Pr 3) with Pr—N distances varying from 2.310 (11) to 3.053 (2) Å. The geometric parameters of Pr_5_Si_3_N_9_ are in the usual ranges and correspond with those of the isotypic compounds Ln_5_Si_3_N_9_ (Ln = La, Ce) and other nitridosilicates (Schnick, 2001; Lissner & Schleid, 2004).

### Experimental

Pr_5_Si_3_N_9_ was synthesized by the reaction of Pr (swarf, 99.9%, Chempur, Karlsruhe) and silicon diimide (Schnick & Huppertz, 1997) which were thoroughly mixed in a glove box (Unilab, MBraun). The mixture was heated in a tungsten crucible in a radio-frequency furnace (Schnick *et al.*, 1999) under purified N_2_ up to 1873 K within 1 h. This temperature was retained for 5 h, and the crucible thereafter cooled down to 1073 K in 35 h before quenching to room temperature within 1 h. Pr_5_Si_3_N_9_ could be obtained as air-sensitive dark-yellow crystals with PrN as by-product.

### Refinement

In the final Fourier map the highest peak is 0.19 Å from atom Si1 and the deepest hole is 0.64 Å from atom Pr4.

### Crystal data

**Table:**

Pr_5_Si_3_N_9_	*F*(000) = 3200
*M**_r_* = 914.91	*D*_x_ = 6.520 Mg m^−^^3^
Orthorhombic, *C**m**c**e*	Mo *K*α radiation, λ = 0.71073 Å
Hall symbol: -C 2 bc 2	Cell parameters from 5699 reflections
*a* = 10.512 (2) Å	θ = 2.6–30.5°
*b* = 11.243 (2) Å	µ = 26.01 mm^−^^1^
*c* = 15.773 (3) Å	*T* = 293 K
*V* = 1864.2 (6) Å^3^	Block, yellow
*Z* = 8	0.17 × 0.10 × 0.08 mm

### Data collection

**Table:**

Stoe IPDS diffractometer	1480 independent reflections
Radiation source: fine-focus sealed tube	1133 reflections with *I* > 2σ(*I*)
graphite	*R*_int_ = 0.080
ω scans	θ_max_ = 30.5°, θ_min_ = 2.6°
Absorption correction: multi-scan (*XPREP*; Sheldrick, 2008)	*h* = −12→14
*T*_min_ = 0.040, *T*_max_ = 0.125	*k* = −15→15
9626 measured reflections	*l* = −22→22

### Refinement

**Table:**

Refinement on *F*^2^	Primary atom site location: structure-invariant direct methods
Least-squares matrix: full	Secondary atom site location: difference Fourier map
*R*[*F*^2^ > 2σ(*F*^2^)] = 0.037	*w* = 1/[σ^2^(*F*_o_^2^) + (0.0626*P*)^2^] where *P* = (*F*_o_^2^ + 2*F*_c_^2^)/3
*wR*(*F*^2^) = 0.097	(Δ/σ)_max_ < 0.001
*S* = 0.97	Δρ_max_ = 2.10 e Å^−^^3^
1480 reflections	Δρ_min_ = −2.31 e Å^−^^3^
90 parameters	Extinction correction: *SHELXL97* (Sheldrick, 2008), Fc^*^=kFc[1+0.001xFc^2^λ^3^/sin(2θ)]^-1/4^
0 restraints	Extinction coefficient: 0.00090 (6)

### Special details

**Table:**

Geometry. All e.s.d.'s (except the e.s.d. in the dihedral angle between two l.s. planes) are estimated using the full covariance matrix. The cell e.s.d.'s are taken into account individually in the estimation of e.s.d.'s in distances, angles and torsion angles; correlations between e.s.d.'s in cell parameters are only used when they are defined by crystal symmetry. An approximate (isotropic) treatment of cell e.s.d.'s is used for estimating e.s.d.'s involving l.s. planes.
Refinement. Refinement of *F*^2^ against ALL reflections. The weighted *R*-factor *wR* and goodness of fit *S* are based on *F*^2^, conventional *R*-factors *R* are based on *F*, with *F* set to zero for negative *F*^2^. The threshold expression of *F*^2^ > σ(*F*^2^) is used only for calculating *R*-factors(gt) *etc*. and is not relevant to the choice of reflections for refinement. *R*-factors based on *F*^2^ are statistically about twice as large as those based on *F*, and *R*- factors based on ALL data will be even larger.

### Fractional atomic coordinates and isotropic or equivalent isotropic displacement parameters (Å^2^)

**Table:**

	*x*	*y*	*z*	*U*_iso_*/*U*_eq_	
Pr1	0.0000	0.01304 (6)	0.33611 (4)	0.01605 (18)	
Pr2	−0.21211 (5)	−0.25683 (4)	0.37015 (3)	0.01847 (17)	
Pr3	0.0000	−0.00567 (6)	0.11240 (4)	0.01844 (19)	
Pr4	0.20546 (7)	0.0000	0.5000	0.01756 (19)	
Si1	0.0000	−0.2598 (3)	0.5166 (2)	0.0148 (6)	
Si2	0.0000	0.2724 (3)	0.2739 (2)	0.0148 (6)	
Si3	−0.2500	−0.0361 (3)	0.2500	0.0146 (6)	
N1	0.0000	0.1552 (10)	0.2035 (7)	0.020 (2)	
N2	0.0000	0.2233 (10)	0.3761 (6)	0.024 (2)	
N3	0.1254 (8)	−0.1348 (7)	0.2416 (5)	0.0201 (14)	
N4	0.2371 (7)	0.0349 (7)	0.3472 (5)	0.0176 (13)	
N5	−0.1321 (8)	−0.3528 (8)	0.5006 (5)	0.0226 (15)	
N6	0.0000	−0.1350 (10)	0.4519 (7)	0.022 (2)	

### Atomic displacement parameters (Å^2^)

**Table:**

	*U*^11^	*U*^22^	*U*^33^	*U*^12^	*U*^13^	*U*^23^
Pr1	0.0137 (3)	0.0145 (3)	0.0199 (3)	0.000	0.000	−0.0005 (2)
Pr2	0.0190 (3)	0.0167 (3)	0.0197 (3)	−0.00032 (16)	−0.00156 (15)	0.00234 (15)
Pr3	0.0220 (4)	0.0160 (3)	0.0173 (3)	0.000	0.000	−0.0013 (2)
Pr4	0.0173 (4)	0.0177 (3)	0.0177 (3)	0.000	0.000	0.0001 (2)
Si1	0.0176 (16)	0.0136 (13)	0.0133 (12)	0.000	0.000	0.0007 (10)
Si2	0.0120 (15)	0.0141 (14)	0.0184 (14)	0.000	0.000	−0.0014 (10)
Si3	0.0113 (14)	0.0164 (14)	0.0160 (14)	0.000	0.0011 (10)	0.000
N1	0.020 (6)	0.019 (4)	0.022 (5)	0.000	0.000	−0.005 (4)
N2	0.041 (7)	0.018 (5)	0.012 (4)	0.000	0.000	−0.002 (3)
N3	0.013 (3)	0.020 (3)	0.027 (4)	−0.001 (3)	0.004 (3)	−0.003 (3)
N4	0.013 (3)	0.024 (3)	0.016 (3)	−0.005 (3)	0.001 (2)	−0.001 (3)
N5	0.019 (4)	0.024 (4)	0.024 (3)	−0.004 (3)	−0.007 (3)	0.005 (3)
N6	0.018 (6)	0.024 (5)	0.024 (5)	0.000	0.000	0.001 (4)

### Geometric parameters (Å, °)

**Table:**

Pr1—N2	2.446 (12)	Pr4—Pr2^xii^	3.5243 (7)
Pr1—N3^i^	2.593 (8)	Pr4—Pr2^i^	3.5408 (7)
Pr1—N3	2.593 (8)	Pr4—Pr2^xv^	3.5408 (7)
Pr1—N6	2.471 (12)	Si1—N6	1.735 (12)
Pr1—N4	2.511 (8)	Si1—N2^xv^	1.742 (10)
Pr1—N4^i^	2.511 (8)	Si1—N5	1.756 (8)
Pr1—N1	2.633 (11)	Si1—N5^i^	1.756 (8)
Pr1—Si3	3.0093 (8)	Si1—Pr3^xvi^	3.039 (3)
Pr1—Si3^ii^	3.0093 (8)	Si1—Pr2^i^	3.211 (2)
Pr1—Si2	3.077 (3)	Si1—Pr3^iii^	3.432 (3)
Pr1—Si2^iii^	3.214 (3)	Si2—N2	1.704 (11)
Pr1—Pr4	3.3717 (8)	Si2—N1	1.723 (11)
Pr2—N3^i^	2.613 (8)	Si2—N3^ix^	1.699 (8)
Pr2—N4^iv^	2.429 (8)	Si2—N3^x^	1.699 (8)
Pr2—N5	2.470 (8)	Si2—Pr3^ix^	3.073 (3)
Pr2—N1^iii^	2.702 (6)	Si2—Pr2^ix^	3.200 (2)
Pr2—N5^v^	2.891 (9)	Si2—Pr2^x^	3.200 (2)
Pr2—N6	2.917 (8)	Si2—Pr1^ix^	3.214 (3)
Pr2—N3^vi^	2.812 (8)	Si2—Pr2^xiii^	3.4018 (16)
Pr2—Si3	3.148 (3)	Si2—Pr2^xvii^	3.4018 (16)
Pr2—Si2^iii^	3.200 (2)	Si3—N3^vi^	1.722 (8)
Pr2—Si1	3.211 (2)	Si3—N3^i^	1.722 (8)
Pr2—Si2^iv^	3.4018 (16)	Si3—N4^vi^	1.733 (7)
Pr2—Pr4^iv^	3.5243 (7)	Si3—N4^i^	1.733 (7)
Pr3—N1	2.310 (11)	Si3—Pr1^vi^	3.0093 (8)
Pr3—N5^vii^	2.752 (8)	Si3—Pr2^xviii^	3.148 (3)
Pr3—N5^viii^	2.752 (8)	Si3—Pr3^vi^	3.4254 (7)
Pr3—N5^ix^	2.839 (9)	N1—Pr2^ix^	2.702 (6)
Pr3—N5^x^	2.839 (9)	N1—Pr2^x^	2.702 (6)
Pr3—N4^xi^	2.873 (8)	N2—Si1^xv^	1.742 (10)
Pr3—N4^vi^	2.873 (8)	N3—Si3^ii^	1.722 (8)
Pr3—Si1^viii^	3.039 (3)	N3—Si2^iii^	1.699 (8)
Pr3—Si2^iii^	3.073 (3)	N3—Pr2^i^	2.613 (8)
Pr3—Si3^ii^	3.4254 (7)	N3—Pr2^ii^	2.812 (8)
Pr3—Si3	3.4254 (7)	N4—Si3^ii^	1.733 (7)
Pr3—Si1^ix^	3.432 (3)	N4—Pr2^xiii^	2.429 (8)
Pr4—N5^xii^	2.378 (8)	N4—Pr3^ii^	2.873 (8)
Pr4—N5^xiii^	2.378 (8)	N5—Pr4^iv^	2.378 (8)
Pr4—N4	2.465 (7)	N5—Pr3^xvi^	2.752 (8)
Pr4—N4^xiv^	2.465 (7)	N5—Pr3^iii^	2.839 (9)
Pr4—N6	2.747 (7)	N5—Pr2^v^	2.891 (9)
Pr4—N6^xv^	2.747 (7)	N6—Pr4^xv^	2.747 (7)
Pr4—Pr1^xv^	3.3717 (8)	N6—Pr2^i^	2.917 (8)
Pr4—Pr2^xiii^	3.5243 (7)		
			
N2—Pr1—N3^i^	140.1 (2)	N4—Pr4—Pr2^xiii^	43.52 (18)
N2—Pr1—N3	140.1 (2)	N4^xiv^—Pr4—Pr2^xiii^	131.21 (19)
N3^i^—Pr1—N3	61.1 (4)	N6—Pr4—Pr2^xiii^	117.5 (2)
N2—Pr1—N6	117.4 (4)	N6^xv^—Pr4—Pr2^xiii^	85.7 (2)
N3^i^—Pr1—N6	89.6 (3)	Pr1—Pr4—Pr2^xiii^	71.228 (16)
N3—Pr1—N6	89.6 (3)	Pr1^xv^—Pr4—Pr2^xiii^	129.553 (18)
N2—Pr1—N4	83.53 (18)	N5^xii^—Pr4—Pr2^xii^	44.41 (19)
N3^i^—Pr1—N4	127.4 (3)	N5^xiii^—Pr4—Pr2^xii^	111.17 (19)
N3—Pr1—N4	66.3 (3)	N4—Pr4—Pr2^xii^	131.21 (19)
N6—Pr1—N4	90.85 (18)	N4^xiv^—Pr4—Pr2^xii^	43.52 (18)
N2—Pr1—N4^i^	83.53 (18)	N6—Pr4—Pr2^xii^	85.7 (2)
N3^i^—Pr1—N4^i^	66.3 (3)	N6^xv^—Pr4—Pr2^xii^	117.5 (2)
N3—Pr1—N4^i^	127.4 (3)	Pr1—Pr4—Pr2^xii^	129.553 (18)
N6—Pr1—N4^i^	90.85 (18)	Pr1^xv^—Pr4—Pr2^xii^	71.228 (16)
N4—Pr1—N4^i^	166.2 (3)	Pr2^xiii^—Pr4—Pr2^xii^	151.53 (3)
N2—Pr1—N1	67.5 (3)	N5^xii^—Pr4—Pr2^i^	54.3 (2)
N3^i^—Pr1—N1	86.1 (3)	N5^xiii^—Pr4—Pr2^i^	123.7 (2)
N3—Pr1—N1	86.1 (3)	N4—Pr4—Pr2^i^	64.00 (18)
N6—Pr1—N1	175.0 (4)	N4^xiv^—Pr4—Pr2^i^	115.66 (18)
N4—Pr1—N1	89.75 (17)	N6—Pr4—Pr2^i^	53.49 (19)
N4^i^—Pr1—N1	89.75 (17)	N6^xv^—Pr4—Pr2^i^	128.75 (19)
N2—Pr1—Si3	107.07 (11)	Pr1—Pr4—Pr2^i^	66.712 (16)
N3^i^—Pr1—Si3	34.80 (19)	Pr1^xv^—Pr4—Pr2^i^	114.875 (19)
N3—Pr1—Si3	93.83 (19)	Pr2^xiii^—Pr4—Pr2^i^	106.955 (19)
N6—Pr1—Si3	102.12 (13)	Pr2^xii^—Pr4—Pr2^i^	72.463 (19)
N4—Pr1—Si3	156.38 (16)	N5^xii^—Pr4—Pr2^xv^	123.7 (2)
N4^i^—Pr1—Si3	35.15 (17)	N5^xiii^—Pr4—Pr2^xv^	54.3 (2)
N1—Pr1—Si3	75.69 (11)	N4—Pr4—Pr2^xv^	115.66 (18)
N2—Pr1—Si3^ii^	107.07 (11)	N4^xiv^—Pr4—Pr2^xv^	64.00 (18)
N3^i^—Pr1—Si3^ii^	93.83 (19)	N6—Pr4—Pr2^xv^	128.75 (19)
N3—Pr1—Si3^ii^	34.80 (19)	N6^xv^—Pr4—Pr2^xv^	53.49 (19)
N6—Pr1—Si3^ii^	102.12 (13)	Pr1—Pr4—Pr2^xv^	114.875 (19)
N4—Pr1—Si3^ii^	35.15 (17)	Pr1^xv^—Pr4—Pr2^xv^	66.712 (16)
N4^i^—Pr1—Si3^ii^	156.38 (16)	Pr2^xiii^—Pr4—Pr2^xv^	72.463 (19)
N1—Pr1—Si3^ii^	75.69 (11)	Pr2^xii^—Pr4—Pr2^xv^	106.955 (19)
Si3—Pr1—Si3^ii^	121.68 (5)	Pr2^i^—Pr4—Pr2^xv^	177.74 (3)
N2—Pr1—Si2	33.5 (2)	N6—Si1—N2^xv^	112.4 (6)
N3^i^—Pr1—Si2	115.08 (18)	N6—Si1—N5	113.4 (3)
N3—Pr1—Si2	115.08 (18)	N2^xv^—Si1—N5	106.3 (4)
N6—Pr1—Si2	150.9 (3)	N6—Si1—N5^i^	113.4 (3)
N4—Pr1—Si2	85.94 (18)	N2^xv^—Si1—N5^i^	106.3 (4)
N4^i^—Pr1—Si2	85.94 (18)	N5—Si1—N5^i^	104.5 (6)
N1—Pr1—Si2	34.0 (2)	N6—Si1—Pr3^xvi^	173.8 (4)
Si3—Pr1—Si2	91.71 (7)	N2^xv^—Si1—Pr3^xvi^	73.8 (4)
Si3^ii^—Pr1—Si2	91.71 (7)	N5—Si1—Pr3^xvi^	63.6 (3)
N2—Pr1—Si2^iii^	162.3 (2)	N5^i^—Si1—Pr3^xvi^	63.6 (3)
N3^i^—Pr1—Si2^iii^	31.79 (18)	N6—Si1—Pr2	64.4 (3)
N3—Pr1—Si2^iii^	31.79 (18)	N2^xv^—Si1—Pr2	134.16 (11)
N6—Pr1—Si2^iii^	80.3 (3)	N5—Si1—Pr2	49.7 (3)
N4—Pr1—Si2^iii^	96.89 (17)	N5^i^—Si1—Pr2	116.8 (3)
N4^i^—Pr1—Si2^iii^	96.89 (17)	Pr3^xvi^—Si1—Pr2	111.53 (8)
N1—Pr1—Si2^iii^	94.7 (2)	N6—Si1—Pr2^i^	64.4 (3)
Si3—Pr1—Si2^iii^	66.54 (6)	N2^xv^—Si1—Pr2^i^	134.16 (11)
Si3^ii^—Pr1—Si2^iii^	66.54 (6)	N5—Si1—Pr2^i^	116.8 (3)
Si2—Pr1—Si2^iii^	128.72 (3)	N5^i^—Si1—Pr2^i^	49.7 (3)
N2—Pr1—Pr4	81.05 (18)	Pr3^xvi^—Si1—Pr2^i^	111.53 (8)
N3^i^—Pr1—Pr4	137.62 (17)	Pr2—Si1—Pr2^i^	87.97 (8)
N3—Pr1—Pr4	95.02 (19)	N6—Si1—Pr3^iii^	107.6 (4)
N6—Pr1—Pr4	53.42 (16)	N2^xv^—Si1—Pr3^iii^	140.0 (4)
N4—Pr1—Pr4	46.78 (16)	N5—Si1—Pr3^iii^	55.6 (3)
N4^i^—Pr1—Pr4	125.95 (16)	N5^i^—Si1—Pr3^iii^	55.6 (3)
N1—Pr1—Pr4	129.46 (13)	Pr3^xvi^—Si1—Pr3^iii^	66.18 (7)
Si3—Pr1—Pr4	153.83 (4)	Pr2—Si1—Pr3^iii^	65.29 (6)
Si3^ii^—Pr1—Pr4	77.212 (18)	Pr2^i^—Si1—Pr3^iii^	65.29 (6)
Si2—Pr1—Pr4	106.60 (5)	N2—Si2—N1	111.2 (6)
Si2^iii^—Pr1—Pr4	112.17 (4)	N2—Si2—N3^ix^	109.6 (4)
N3^i^—Pr2—N4^iv^	117.8 (2)	N1—Si2—N3^ix^	112.2 (4)
N3^i^—Pr2—N5	139.2 (3)	N2—Si2—N3^x^	109.6 (4)
N4^iv^—Pr2—N5	77.2 (3)	N1—Si2—N3^x^	112.2 (4)
N3^i^—Pr2—N1^iii^	64.6 (3)	N3^ix^—Si2—N3^x^	101.8 (6)
N4^iv^—Pr2—N1^iii^	76.4 (3)	N2—Si2—Pr3^ix^	73.2 (4)
N5—Pr2—N1^iii^	85.3 (3)	N1—Si2—Pr3^ix^	175.6 (4)
N3^i^—Pr2—N5^v^	121.4 (2)	N3^ix^—Si2—Pr3^ix^	65.5 (3)
N4^iv^—Pr2—N5^v^	113.1 (2)	N3^x^—Si2—Pr3^ix^	65.5 (3)
N5—Pr2—N5^v^	78.0 (3)	N2—Si2—Pr1	52.5 (4)
N1^iii^—Pr2—N5^v^	157.9 (3)	N1—Si2—Pr1	58.7 (4)
N3^i^—Pr2—N6	80.2 (3)	N3^ix^—Si2—Pr1	128.9 (3)
N4^iv^—Pr2—N6	133.4 (3)	N3^x^—Si2—Pr1	128.9 (3)
N5—Pr2—N6	65.0 (3)	Pr3^ix^—Si2—Pr1	125.69 (11)
N1^iii^—Pr2—N6	74.4 (3)	N2—Si2—Pr2^ix^	129.66 (18)
N5^v^—Pr2—N6	85.5 (2)	N1—Si2—Pr2^ix^	57.6 (2)
N3^i^—Pr2—N3^vi^	57.9 (3)	N3^ix^—Si2—Pr2^ix^	120.1 (3)
N4^iv^—Pr2—N3^vi^	104.1 (2)	N3^x^—Si2—Pr2^ix^	54.6 (3)
N5—Pr2—N3^vi^	160.4 (3)	Pr3^ix^—Si2—Pr2^ix^	119.86 (7)
N1^iii^—Pr2—N3^vi^	114.2 (3)	Pr1—Si2—Pr2^ix^	97.41 (8)
N5^v^—Pr2—N3^vi^	83.7 (2)	N2—Si2—Pr2^x^	129.66 (18)
N6—Pr2—N3^vi^	120.8 (3)	N1—Si2—Pr2^x^	57.6 (2)
N3^i^—Pr2—Si3	33.16 (18)	N3^ix^—Si2—Pr2^x^	54.6 (3)
N4^iv^—Pr2—Si3	129.97 (18)	N3^x^—Si2—Pr2^x^	120.1 (3)
N5—Pr2—Si3	152.7 (2)	Pr3^ix^—Si2—Pr2^x^	119.86 (7)
N1^iii^—Pr2—Si3	97.7 (2)	Pr1—Si2—Pr2^x^	97.41 (8)
N5^v^—Pr2—Si3	90.97 (16)	Pr2^ix^—Si2—Pr2^x^	88.33 (8)
N6—Pr2—Si3	89.6 (2)	N2—Si2—Pr1^ix^	141.6 (4)
N3^vi^—Pr2—Si3	32.97 (16)	N1—Si2—Pr1^ix^	107.2 (4)
N3^i^—Pr2—Si2^iii^	32.00 (18)	N3^ix^—Si2—Pr1^ix^	53.5 (3)
N4^iv^—Pr2—Si2^iii^	98.41 (18)	N3^x^—Si2—Pr1^ix^	53.5 (3)
N5—Pr2—Si2^iii^	113.5 (2)	Pr3^ix^—Si2—Pr1^ix^	68.39 (7)
N1^iii^—Pr2—Si2^iii^	32.6 (2)	Pr1—Si2—Pr1^ix^	165.93 (11)
N5^v^—Pr2—Si2^iii^	148.38 (18)	Pr2^ix^—Si2—Pr1^ix^	72.74 (6)
N6—Pr2—Si2^iii^	74.5 (2)	Pr2^x^—Si2—Pr1^ix^	72.74 (6)
N3^vi^—Pr2—Si2^iii^	85.88 (18)	N2—Si2—Pr2^xiii^	63.03 (5)
Si3—Pr2—Si2^iii^	65.15 (5)	N1—Si2—Pr2^xiii^	102.34 (17)
N3^i^—Pr2—Si1	108.77 (18)	N3^ix^—Si2—Pr2^xiii^	55.5 (3)
N4^iv^—Pr2—Si1	104.47 (19)	N3^x^—Si2—Pr2^xiii^	144.5 (3)
N5—Pr2—Si1	32.8 (2)	Pr3^ix^—Si2—Pr2^xiii^	79.51 (6)
N1^iii^—Pr2—Si1	74.5 (2)	Pr1—Si2—Pr2^xiii^	76.47 (6)
N5^v^—Pr2—Si1	83.71 (16)	Pr2^ix^—Si2—Pr2^xiii^	157.94 (9)
N6—Pr2—Si1	32.5 (2)	Pr2^x^—Si2—Pr2^xiii^	71.77 (2)
N3^vi^—Pr2—Si1	151.39 (17)	Pr1^ix^—Si2—Pr2^xiii^	108.81 (6)
Si3—Pr2—Si1	121.96 (6)	N2—Si2—Pr2^xvii^	63.03 (5)
Si2^iii^—Pr2—Si1	91.60 (6)	N1—Si2—Pr2^xvii^	102.34 (17)
N3^i^—Pr2—Si2^iv^	85.02 (18)	N3^ix^—Si2—Pr2^xvii^	144.5 (3)
N4^iv^—Pr2—Si2^iv^	80.27 (19)	N3^x^—Si2—Pr2^xvii^	55.5 (3)
N5—Pr2—Si2^iv^	135.8 (2)	Pr3^ix^—Si2—Pr2^xvii^	79.51 (6)
N1^iii^—Pr2—Si2^iv^	125.3 (2)	Pr1—Si2—Pr2^xvii^	76.47 (6)
N5^v^—Pr2—Si2^iv^	76.68 (16)	Pr2^ix^—Si2—Pr2^xvii^	71.77 (2)
N6—Pr2—Si2^iv^	146.3 (2)	Pr2^x^—Si2—Pr2^xvii^	157.94 (9)
N3^vi^—Pr2—Si2^iv^	29.85 (17)	Pr1^ix^—Si2—Pr2^xvii^	108.81 (6)
Si3—Pr2—Si2^iv^	62.78 (6)	Pr2^xiii^—Si2—Pr2^xvii^	125.65 (10)
Si2^iii^—Pr2—Si2^iv^	107.04 (3)	N3^vi^—Si3—N3^i^	99.8 (6)
Si1—Pr2—Si2^iv^	160.04 (7)	N3^vi^—Si3—N4^vi^	107.8 (4)
N3^i^—Pr2—Pr4^iv^	160.79 (17)	N3^i^—Si3—N4^vi^	106.7 (4)
N4^iv^—Pr2—Pr4^iv^	44.35 (17)	N3^vi^—Si3—N4^i^	106.7 (4)
N5—Pr2—Pr4^iv^	42.36 (19)	N3^i^—Si3—N4^i^	107.8 (4)
N1^iii^—Pr2—Pr4^iv^	99.7 (2)	N4^vi^—Si3—N4^i^	125.2 (5)
N5^v^—Pr2—Pr4^iv^	77.38 (17)	N3^vi^—Si3—Pr1	138.4 (3)
N6—Pr2—Pr4^iv^	107.2 (2)	N3^i^—Si3—Pr1	59.3 (3)
N3^vi^—Pr2—Pr4^iv^	126.40 (17)	N4^vi^—Si3—Pr1	112.5 (3)
Si3—Pr2—Pr4^iv^	158.49 (2)	N4^i^—Si3—Pr1	56.5 (3)
Si2^iii^—Pr2—Pr4^iv^	131.58 (6)	N3^vi^—Si3—Pr1^vi^	59.3 (3)
Si1—Pr2—Pr4^iv^	75.19 (5)	N3^i^—Si3—Pr1^vi^	138.4 (3)
Si2^iv^—Pr2—Pr4^iv^	96.64 (5)	N4^vi^—Si3—Pr1^vi^	56.5 (3)
N1—Pr3—N5^vii^	148.38 (19)	N4^i^—Si3—Pr1^vi^	112.5 (3)
N1—Pr3—N5^viii^	148.38 (19)	Pr1—Si3—Pr1^vi^	158.86 (12)
N5^vii^—Pr3—N5^viii^	60.6 (3)	N3^vi^—Si3—Pr2^xviii^	56.1 (3)
N1—Pr3—N5^ix^	85.2 (3)	N3^i^—Si3—Pr2^xviii^	62.7 (3)
N5^vii^—Pr3—N5^ix^	72.6 (3)	N4^vi^—Si3—Pr2^xviii^	79.7 (3)
N5^viii^—Pr3—N5^ix^	101.2 (2)	N4^i^—Si3—Pr2^xviii^	154.9 (3)
N1—Pr3—N5^x^	85.2 (3)	Pr1—Si3—Pr2^xviii^	121.79 (7)
N5^vii^—Pr3—N5^x^	101.2 (2)	Pr1^vi^—Si3—Pr2^xviii^	76.26 (3)
N5^viii^—Pr3—N5^x^	72.6 (3)	N3^vi^—Si3—Pr2	62.7 (3)
N5^ix^—Pr3—N5^x^	58.6 (3)	N3^i^—Si3—Pr2	56.1 (3)
N1—Pr3—N4^xi^	74.78 (15)	N4^vi^—Si3—Pr2	154.9 (3)
N5^vii^—Pr3—N4^xi^	136.0 (2)	N4^i^—Si3—Pr2	79.7 (3)
N5^viii^—Pr3—N4^xi^	75.5 (2)	Pr1—Si3—Pr2	76.26 (3)
N5^ix^—Pr3—N4^xi^	120.9 (2)	Pr1^vi^—Si3—Pr2	121.79 (7)
N5^x^—Pr3—N4^xi^	64.7 (2)	Pr2^xviii^—Si3—Pr2	75.92 (8)
N1—Pr3—N4^vi^	74.78 (15)	N3^vi^—Si3—Pr3	134.2 (3)
N5^vii^—Pr3—N4^vi^	75.5 (2)	N3^i^—Si3—Pr3	55.4 (3)
N5^viii^—Pr3—N4^vi^	136.0 (2)	N4^vi^—Si3—Pr3	56.9 (3)
N5^ix^—Pr3—N4^vi^	64.7 (2)	N4^i^—Si3—Pr3	117.0 (3)
N5^x^—Pr3—N4^vi^	120.9 (2)	Pr1—Si3—Pr3	66.28 (2)
N4^xi^—Pr3—N4^vi^	148.3 (3)	Pr1^vi^—Si3—Pr3	111.45 (3)
N1—Pr3—Si1^viii^	171.4 (3)	Pr2^xviii^—Si3—Pr3	78.13 (3)
N5^vii^—Pr3—Si1^viii^	34.86 (17)	Pr2—Si3—Pr3	111.28 (6)
N5^viii^—Pr3—Si1^viii^	34.86 (17)	N3^vi^—Si3—Pr3^vi^	55.4 (3)
N5^ix^—Pr3—Si1^viii^	102.31 (17)	N3^i^—Si3—Pr3^vi^	134.2 (3)
N5^x^—Pr3—Si1^viii^	102.31 (17)	N4^vi^—Si3—Pr3^vi^	117.0 (3)
N4^xi^—Pr3—Si1^viii^	104.38 (15)	N4^i^—Si3—Pr3^vi^	56.9 (3)
N4^vi^—Pr3—Si1^viii^	104.38 (15)	Pr1—Si3—Pr3^vi^	111.45 (3)
N1—Pr3—Si2^iii^	105.8 (3)	Pr1^vi^—Si3—Pr3^vi^	66.28 (2)
N5^vii^—Pr3—Si2^iii^	84.53 (19)	Pr2^xviii^—Si3—Pr3^vi^	111.28 (6)
N5^viii^—Pr3—Si2^iii^	84.53 (19)	Pr2—Si3—Pr3^vi^	78.13 (3)
N5^ix^—Pr3—Si2^iii^	149.10 (17)	Pr3—Si3—Pr3^vi^	168.55 (11)
N5^x^—Pr3—Si2^iii^	149.10 (17)	Si2—N1—Pr3	178.3 (7)
N4^xi^—Pr3—Si2^iii^	89.97 (15)	Si2—N1—Pr1	87.3 (5)
N4^vi^—Pr3—Si2^iii^	89.97 (15)	Pr3—N1—Pr1	91.1 (4)
Si1^viii^—Pr3—Si2^iii^	65.53 (9)	Si2—N1—Pr2^ix^	89.8 (3)
N1—Pr3—Si3^ii^	71.58 (17)	Pr3—N1—Pr2^ix^	91.1 (3)
N5^vii^—Pr3—Si3^ii^	137.36 (18)	Pr1—N1—Pr2^ix^	124.3 (2)
N5^viii^—Pr3—Si3^ii^	87.79 (18)	Si2—N1—Pr2^x^	89.8 (3)
N5^ix^—Pr3—Si3^ii^	146.45 (18)	Pr3—N1—Pr2^x^	91.1 (3)
N5^x^—Pr3—Si3^ii^	94.75 (16)	Pr1—N1—Pr2^x^	124.3 (2)
N4^xi^—Pr3—Si3^ii^	30.36 (15)	Pr2^ix^—N1—Pr2^x^	111.2 (4)
N4^vi^—Pr3—Si3^ii^	127.82 (14)	Si2—N2—Si1^xv^	147.4 (8)
Si1^viii^—Pr3—Si3^ii^	103.21 (6)	Si2—N2—Pr1	94.0 (4)
Si2^iii^—Pr3—Si3^ii^	63.20 (6)	Si1^xv^—N2—Pr1	118.6 (6)
N1—Pr3—Si3	71.58 (17)	Si3^ii^—N3—Si2^iii^	175.7 (6)
N5^vii^—Pr3—Si3	87.79 (18)	Si3^ii^—N3—Pr2^i^	90.7 (4)
N5^viii^—Pr3—Si3	137.36 (18)	Si2^iii^—N3—Pr2^i^	93.4 (3)
N5^ix^—Pr3—Si3	94.75 (16)	Si3^ii^—N3—Pr1	85.9 (3)
N5^x^—Pr3—Si3	146.45 (18)	Si2^iii^—N3—Pr1	94.7 (3)
N4^xi^—Pr3—Si3	127.82 (14)	Pr2^i^—N3—Pr1	93.9 (3)
N4^vi^—Pr3—Si3	30.36 (15)	Si3^ii^—N3—Pr2^ii^	84.3 (3)
Si1^viii^—Pr3—Si3	103.21 (6)	Si2^iii^—N3—Pr2^ii^	94.7 (3)
Si2^iii^—Pr3—Si3	63.20 (6)	Pr2^i^—N3—Pr2^ii^	91.0 (2)
Si3^ii^—Pr3—Si3	100.21 (3)	Pr1—N3—Pr2^ii^	169.1 (3)
N1—Pr3—Si1^ix^	74.8 (3)	Si3^ii^—N4—Pr2^xiii^	124.0 (4)
N5^vii^—Pr3—Si1^ix^	94.91 (19)	Si3^ii^—N4—Pr4	143.3 (4)
N5^viii^—Pr3—Si1^ix^	94.91 (19)	Pr2^xiii^—N4—Pr4	92.1 (2)
N5^ix^—Pr3—Si1^ix^	30.70 (16)	Si3^ii^—N4—Pr1	88.3 (3)
N5^x^—Pr3—Si1^ix^	30.70 (16)	Pr2^xiii^—N4—Pr1	108.9 (3)
N4^xi^—Pr3—Si1^ix^	90.21 (15)	Pr4—N4—Pr1	85.3 (2)
N4^vi^—Pr3—Si1^ix^	90.21 (15)	Si3^ii^—N4—Pr3^ii^	92.7 (3)
Si1^viii^—Pr3—Si1^ix^	113.82 (7)	Pr2^xiii^—N4—Pr3^ii^	84.8 (2)
Si2^iii^—Pr3—Si1^ix^	179.35 (8)	Pr4—N4—Pr3^ii^	83.5 (2)
Si3^ii^—Pr3—Si1^ix^	117.13 (6)	Pr1—N4—Pr3^ii^	162.7 (3)
Si3—Pr3—Si1^ix^	117.13 (6)	Si1—N5—Pr4^iv^	169.2 (5)
N5^xii^—Pr4—N5^xiii^	88.2 (4)	Si1—N5—Pr2	97.4 (4)
N5^xii^—Pr4—N4	90.6 (3)	Pr4^iv^—N5—Pr2	93.2 (3)
N5^xiii^—Pr4—N4	78.2 (3)	Si1—N5—Pr3^xvi^	81.5 (3)
N5^xii^—Pr4—N4^xiv^	78.2 (3)	Pr4^iv^—N5—Pr3^xvi^	87.8 (3)
N5^xiii^—Pr4—N4^xiv^	90.6 (3)	Pr2—N5—Pr3^xvi^	163.4 (4)
N4—Pr4—N4^xiv^	164.5 (4)	Si1—N5—Pr3^iii^	93.7 (4)
N5^xii^—Pr4—N6	100.3 (3)	Pr4^iv^—N5—Pr3^iii^	85.8 (3)
N5^xiii^—Pr4—N6	161.9 (3)	Pr2—N5—Pr3^iii^	84.7 (2)
N4—Pr4—N6	85.6 (3)	Pr3^xvi^—N5—Pr3^iii^	78.8 (2)
N4^xiv^—Pr4—N6	106.7 (3)	Si1—N5—Pr2^v^	95.3 (4)
N5^xii^—Pr4—N6^xv^	161.9 (3)	Pr4^iv^—N5—Pr2^v^	83.8 (3)
N5^xiii^—Pr4—N6^xv^	100.3 (3)	Pr2—N5—Pr2^v^	102.0 (3)
N4—Pr4—N6^xv^	106.7 (3)	Pr3^xvi^—N5—Pr2^v^	94.6 (2)
N4^xiv^—Pr4—N6^xv^	85.6 (3)	Pr3^iii^—N5—Pr2^v^	167.9 (3)
N6—Pr4—N6^xv^	76.3 (4)	Si1—N6—Pr1	168.4 (7)
N5^xii^—Pr4—Pr1	119.2 (2)	Si1—N6—Pr4	106.5 (4)
N5^xiii^—Pr4—Pr1	115.63 (19)	Pr1—N6—Pr4	80.3 (3)
N4—Pr4—Pr1	47.91 (18)	Si1—N6—Pr4^xv^	106.5 (4)
N4^xiv^—Pr4—Pr1	147.47 (18)	Pr1—N6—Pr4^xv^	80.3 (3)
N6—Pr4—Pr1	46.3 (2)	Pr4—N6—Pr4^xv^	103.7 (4)
N6^xv^—Pr4—Pr1	71.6 (2)	Si1—N6—Pr2	83.1 (3)
N5^xii^—Pr4—Pr1^xv^	115.63 (19)	Pr1—N6—Pr2	89.4 (3)
N5^xiii^—Pr4—Pr1^xv^	119.2 (2)	Pr4—N6—Pr2	169.3 (5)
N4—Pr4—Pr1^xv^	147.47 (18)	Pr4^xv^—N6—Pr2	77.32 (4)
N4^xiv^—Pr4—Pr1^xv^	47.91 (18)	Si1—N6—Pr2^i^	83.1 (3)
N6—Pr4—Pr1^xv^	71.6 (2)	Pr1—N6—Pr2^i^	89.4 (3)
N6^xv^—Pr4—Pr1^xv^	46.3 (2)	Pr4—N6—Pr2^i^	77.32 (4)
Pr1—Pr4—Pr1^xv^	100.33 (3)	Pr4^xv^—N6—Pr2^i^	169.3 (5)
N5^xii^—Pr4—Pr2^xiii^	111.17 (19)	Pr2—N6—Pr2^i^	99.7 (3)
N5^xiii^—Pr4—Pr2^xiii^	44.41 (19)		

Symmetry codes: (i) −*x*, *y*, *z*; (ii) *x*+1/2, *y*, −*z*+1/2; (iii) *x*, *y*−1/2, −*z*+1/2; (iv) *x*−1/2, *y*−1/2, *z*; (v) −*x*−1/2, −*y*−1/2, −*z*+1; (vi) *x*−1/2, *y*, −*z*+1/2; (vii) *x*, −*y*−1/2, *z*−1/2; (viii) −*x*, −*y*−1/2, *z*−1/2; (ix) *x*, *y*+1/2, −*z*+1/2; (x) −*x*, *y*+1/2, −*z*+1/2; (xi) −*x*+1/2, *y*, −*z*+1/2; (xii) *x*+1/2, −*y*−1/2, −*z*+1; (xiii) *x*+1/2, *y*+1/2, *z*; (xiv) *x*, −*y*, −*z*+1; (xv) −*x*, −*y*, −*z*+1; (xvi) −*x*, −*y*−1/2, *z*+1/2; (xvii) −*x*−1/2, *y*+1/2, *z*; (xviii) −*x*−1/2, *y*, −*z*+1/2.
